# Light-Triggered Radiochemical Synthesis: A Novel ^18^F-Labelling Strategy Using Photoinducible Click Reaction to Prepare PET Imaging Probes

**DOI:** 10.1155/2018/4617493

**Published:** 2018-06-25

**Authors:** Ji Hae Choi, Doori Oh, In Sun Kim, Hyeon-Soo Kim, Minjoo Kim, Eun-Mi Kim, Seok Tae Lim, Myung-Hee Sohn, Dong Hyun Kim, Hwan-Jeong Jeong

**Affiliations:** ^1^Department of Nuclear Medicine, Molecular Imaging and Therapeutic Medicine Research Center, Chonbuk National University Medical School and Hospital, Jeonju, Jeollabuk-do 54896, Republic of Korea; ^2^Research Institute of Clinical Medicine, Biomedical Research Institute, Chonbuk National University Hospital, Chonbuk National University, Jeonju, Jeollabuk-do 54896, Republic of Korea; ^3^Kaibiotech, 20 Geonji-ro, Deokjin-gu, Jeonju, Jeollabuk-do 54896, Republic of Korea

## Abstract

Novel probe development for positron emission tomography (PET) is leading to expanding the scope of molecular imaging. To begin responding to challenges, several biomaterials such as natural products and small molecules, peptides, engineered proteins including affibodies, and antibodies have been used in the development of targeted molecular imaging probes. To prepare radiotracers, a few bioactive materials are unique challenges to radiolabelling because of their complex structure, poor stability, poor solubility in aqueous or chemical organic solutions, and sensitivity to temperature and nonphysiological pH. To overcome these challenges, we developed a new radiolabelling strategy based on photoactivated 1,3-dipolar cycloaddition between alkene dipolarophile and tetrazole moiety containing compounds. Herein, we describe a light-triggered radiochemical synthesis via photoactivated click reaction to prepare ^18^F-radiolabelled PET tracers using small molecular and RGD peptide.

## 1. Introduction

Molecular imaging probes provide better understanding of fundamental pathways to monitor biochemical changes *in vivo*. They are important for diagnosis, monitoring of therapeutic response, and drug development [1, 2]. PET is an attractive nuclear medicine technique that serves as a noninvasive and functional imaging modality at picomolar levels *in vivo* with excellent sensitivity based on positron-emitting radionuclide [3, 4]. PET scan information can be used to assess biological processes *in vivo* during early stages of various diseases, including cancer, heart disease, and dementia in Alzheimer's disease and Parkinson's disease. It is also important to assess response to chemotherapy or radiotherapy in various malignancies [5, 6]. Several radiopharmaceuticals targeting specific diseases have been developed. Among various PET tracers, FDG (2-deoxy-2-[^18^F]fluoro-D-glucose) is the most commonly used one in nuclear medicine and molecular cellular biology. FDG was discovered approximately 40 years ago [7]. It has led to a new medical paradigm involving more accurate diagnosis via functional information through quantitative analysis in fields of oncology, neuroscience, and cardiology. Several studies on ^18^F-radiolabelled targeting radiotracers for disease monitoring via PET images have been reported. Fluorine-18 has a relatively long half-life (*T*_1/2_ = 109.8 min) and low energy (0.635 MeV) that permits PET imaging protocols with a duration up to 6 h and short positron linear range in tissue due to low positron energy, resulting in high sensitivity in PET imaging [8, 9]. However, ^18^F-incorporation into biotargeting vectors can be challenging because it must be performed rapidly and efficiently under mild radiolabelling conditions due to short half-life of the radioisotope and regioselectivity labelling of high specificity with acceptable radiochemical yield [10]. To overcome these obstacles, ^18^F-radiolabelling strategies using ^18^F-prosthetic groups such as *N*-succinimidyl-4-[^18^F]fluorobenzoate ([^18^F]SFB) [11–15], 2-bromo-*N*-[3-(2-[^18^F]fluoropyridin-3-yloxy)propyl]acetamide ([^18^F]FPyBrA) [16, 17], and *N*-(4-[^18^F]fluorobenzyl)-2-bromoacetamide ([^18^F]FBBA) [18, 19] have been introduced for labelling of amine and sulfhydryl functionalities of sensitive biomolecules, including small molecules, peptides, and proteins. Click chemistry, copper(I)-catalyzed Huisgen 1,3-dipolar cycloaddition, is still one of the attractive approaches to prepare targeted molecular imaging probes for PET by radiolabelling with small molecules, peptides, and proteins. A number of novel radiotracers have been reported through the click chemistry, including copper-free approaches to avoid toxicity to humans who are susceptible to even low levels of copper [20–27]. Recently, Lin and coworkers have reported that advanced photoinducible 1,3-dipolar cycloaddition reactions show extremely rapid reaction rate and high regioselectivity of desired product without opposite regioisomer under photoactivated mild condition [28–30]. This has stimulated efforts to further develop ^18^F-radiolabelling strategy to create a new labelling platform using photoactivation by UV radiation. Herein, we report a new strategy that utilizes photoactivated click chemistry between ^18^F-labelled terminal alkene dipolarophile and tetrazole moiety compound under radiation using UV light source for a biocompatible and mild reaction with significantly high radiochemical yield and molar activity of desired radiolabelled product for PET imaging.

## 2. Results and Discussion

**Scheme 1:**
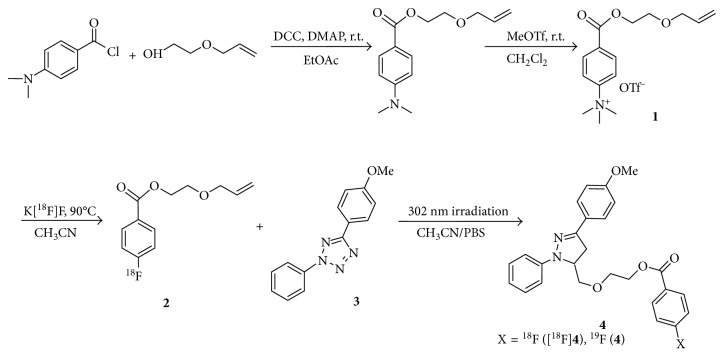
Synthesis of [^18^F]**4** and **4** using photoinducible click reaction.

**Figure 1:**
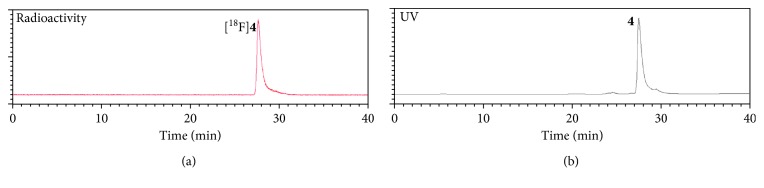
Analytic HPLC profile of [^18^F]**4** (a) with coinjection of the authentic compound **4** (b).

**Figure 2:**
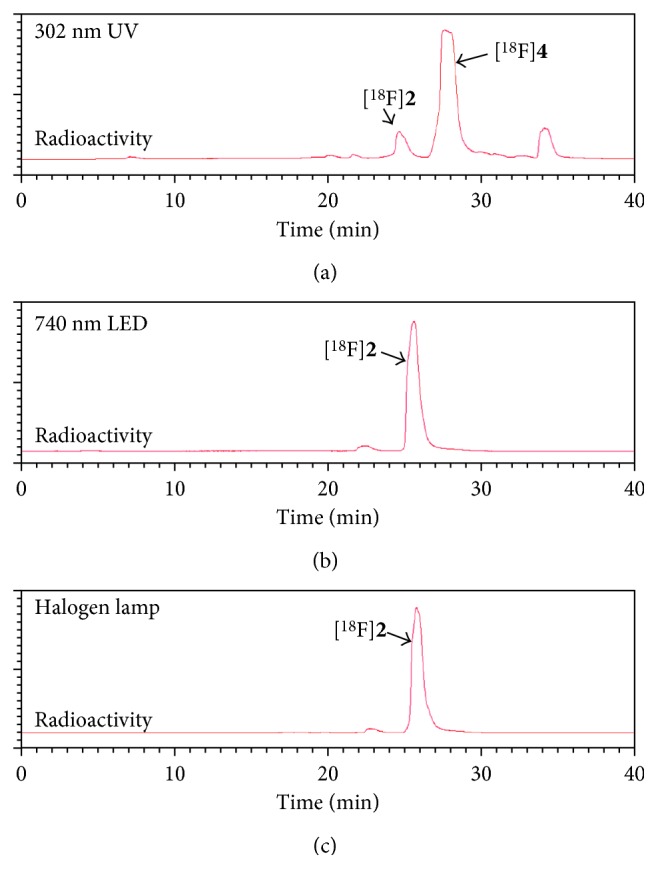
Comparison of light effect for photoinducible click reaction. (a) 302 nm UV lamp, (b) 740 nm LED, and (c) halogen lamp. The desired photoinduced radiolabelled compound [^18^F]**4** was obtained only under irradiation of 302 nm UV (a).

**Scheme 2:**
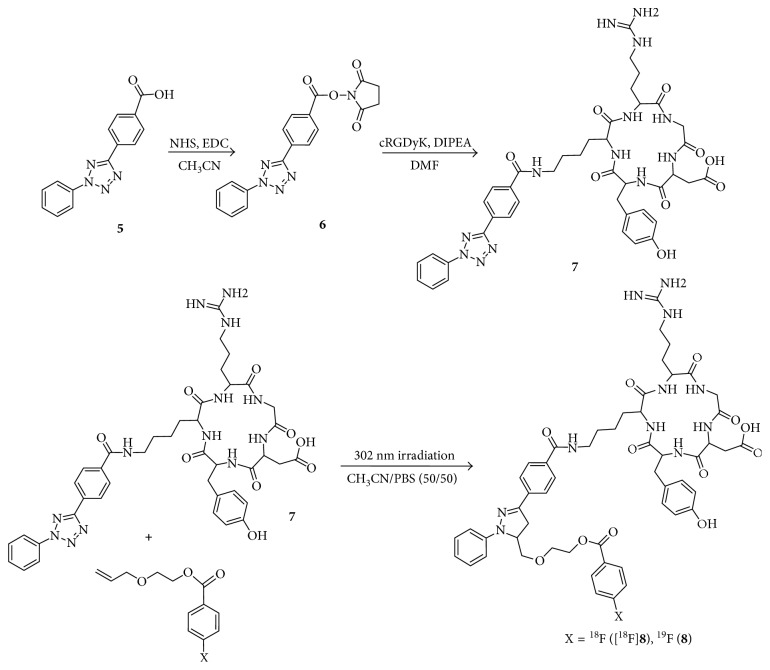
Synthesis of ^18^FB-PEGhc-RGD ([^18^F]**8**) and **8** using photoinducible click reaction.

First, to determine whether photoinducible click reaction with radiolabelled compound could be used as a novel approach to prepare radiopharmaceuticals, we examined photoactivated radiolabelling between ^18^F-radiolabelled compound [^18^F]**2** and 2,5-diaryl tetrazole compound **3** under mild condition using handheld 302 nm UV lamp. For feasibility study of radiochemical synthesis using photoinducible reaction, we chose a 2-(allyloxy)ethanol as a terminal alkene moiety. It is commercially available. It readily undergoes a catalyst-free cycloaddition reaction with photoinduced nitrile imine from tetrazole compound. To prepare ^18^F-radiolabelled compound [^18^F]**2**, trimethylammonium triflate precursor **1** was prepared from 4-(dimethylamino)benzoyl chloride and conjugated with 2-(allyloxy)ethanol followed by conversion of the dimethylamino functional group using methyl triflate at room temperature. Synthesis of 2,5-diaryl tetrazole compound **3** was performed using a previously reported procedure [11]. Radiolabelling of precursor **1** was carried out with K[^18^F]F in CH_3_CN (prepared following azeotropic distillation of a CH_3_CN solution containing K_2_CO_3_ and Kryptofix 2.2.2) at 90°C for 10 min to give 2-(allyloxy)ethyl-4-[^18^F]fluorobenzoate [^18^F]**2** followed by photoinduced cycloaddition with 2,5-diaryl tetrazole compound **3** in situ (Scheme 1) at room temperature under 302 nm UV radiation for 30 min. The resulting radiolabelled compound [^18^F]**2** was obtained with 80% yield as determined by radio-TLC. It was used without purification for subsequent photoinducible click reaction with tetrazole compound **3**. Photo click reaction between ^18^F-radiolabelled terminal alkene compound [^18^F]**2** and tetrazole compound **3** produced the desired product [^18^F]**4**. It was isolated by semipreparative HPLC with an overall yield of 20–27% (decay-corrected, *n*=4). It had high molar activity of 30–112 GBq/*μ*mol (*n*=4) with radiochemical purity >98%. Purified [^18^F]**4** was identified with nonradioactive reference compound **4** by analytical HPLC (Figure 1). Increasing irradiation time to 5–30 min of 302 nm UV lamp resulted in the maximum radiochemical yield at room temperature. In order to investigate the radiochemical yield with reaction time, the best result was obtained at 30 min under the same reaction condition with the time difference of 5 to 30 min. Heating conditions were not considered for the application of temperature-sensitive proteins or peptides. Results indicated that the radiochemical synthesis through photoinducible reaction could be performed under mild conditions, which was possible for both large and small molecules. To evaluate the wavelength effect of light source, we carried out optimized experiment with tetrazole compound **3** and [^18^F]**2** under irradiation of different light sources such as halogen and red LED (720 nm) at room temperature. The desired product [^18^F]**4** was obtained when 302 nm UV light was employed. However, no reaction was observed when halogen or red LED was employed (Figure 2). Of various light sources, only 302 nm UV led to a good conversion to intermediate nitrile imine when 2,5-diaryl tetrazole compound **3** was used. These results demonstrated that photoactivated click reaction between 2-(allyloxy)ethyl-4-[^18^F]fluorobenzoate [^18^F]**2** and nitrile imine was efficient under irradiation with 302 nm UV. Of various factors investigated for photoactivated click reaction, UV wavelength was found to be the most important factor to synthesize the desired product. We aimed to develop water-soluble mass materials such as peptides or proteins as imaging agents. Hence, we subsequently applied the method to radiolabel cyclic RGD peptide and evaluated tumor targeting ability of integrin *α*_v_*β*_3_ as a molecular imaging probe. Integrin *α*_v_*β*_3_ is associated with angiogenic endothelial as well as tumor cells, including cancers of the prostate, skin, ovary, kidney, lung, and breast. RGD peptides can specifically and strongly bind to integrin *α*_v_*β*_3_ [31, 32]. Cyclic RGDyK peptide was selected as targeting material due to its high binding affinity to tumor cells, small size peptide, and available description in the literature on tumor studies. We performed conjugation with 2,5-diaryl tetrazole benzoic acid (**5**) to form 2,5-diaryl tetrazole-RGD (**7**) by reaction of NH_2_-Lys-RGD with *N*-hydroxysuccinimide active ester of 2,5-diaryl tetrazole (Scheme 2). It was purified with HPLC. For radiochemical synthesis of the desired ^18^F-labelled RGD peptide ([^18^F]**8**), photoactivated click reaction was performed between 2,5-diaryl tetrazole-RGD peptide (**7**) and 2-(allyloxy)ethyl-4-[^18^F]fluorobenzoate ([^18^F]**4**) using 302 nm UV lamp for irradiation at room temperature. After 20 min of reaction, the crude mixture was purified with HPLC.

**Figure 3:**
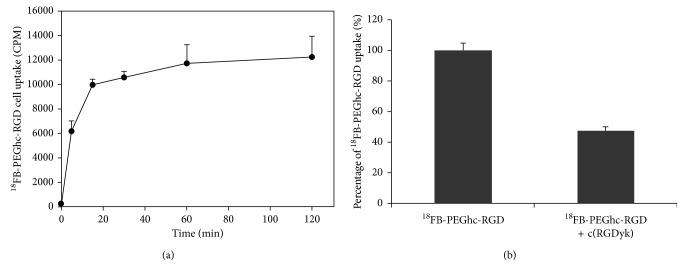
U87MG cell uptake (a) and inhibition study (b) of ^18^FB-PEGHc-RGD ([^18^F]**8**) in U87MG cells. Significant radioactivity accumulation in U87MG cells was observed in the presence of ^18^FB-PEGHc-RGD ([^18^F]**8**). Inhibition study using nonradiolabelled c(RGDyK) showed 52% reduction in cell uptake. Data are expressed as mean ± SD from quadruplicate samples.

Identity of the radiolabelled product ([^18^F]**8**) was confirmed by HPLC retention time after coinjection with authentic nonradioactive compound **8**. Radiochemical synthesis of [^18^F]**8** was successfully carried out by photoactivation. Radiochemical yield from [^18^F]fluoride was 10–12% (*n*=4) by two-step one-pot reaction. The desired product showed excellent purity with adequate molar activities (20–72 GBq/*μ*mol) in a total synthesis time of 95 min (including HPLC purification and reformulation). Based on these results, introducing a 2-(allyloxy)ethyl-4-[^18^F]fluorobenzoate ([^18^F]**2**) tag into peptide is suitable and effective due to the use of aqueous reaction media at room temperature and high chemoselectivity without requiring toxic metal catalyst. Biologically, the uptake of [^18^F]**8** by U87MG tumor cell was increased in a time-dependent manner, plateauing between 60 and 120 min. Furthermore, U87MG cell blocking study using nonradiolabelled c(RGDyK) peptide showed cell uptake inhibition indicative of specific binding of [^18^F]**8** to integrin *α*_v_*β*_3_ expressed in U87MG glioblastoma cells (Figure 3).

**Figure 4:**
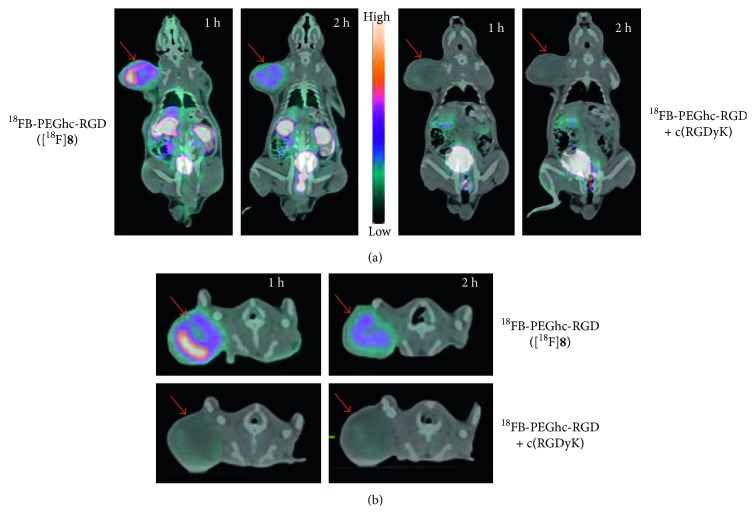
(a) PET/CT images of ^18^FB-PEGHc-RGD ([^18^F]**8**) in RR1022 tumor-bearing rats at 1 h and 2 h after injection. High radioactivity accumulations were found in the tumor (red arrow) at 1 h and 2 h after injection of ^18^FB-PEGHc-RGD ([^18^F]**8**). Inhibition study using nonradiolabelled c(RGDyK) showed complete blocking of radioactivity in the tumor. (b) Transaxial views showing high tumor uptake of ^18^FB-PEGHc-RGD ([^18^F]**8**).

*In vivo* PET imaging following intravenous injection of [^18^F]**8** (4.66 MBq tail-vein injected) to RR1022 tumor-bearing SD rats showed tumor uptake of [^18^F]**8** with renal clearance of ^18^F-activity and high tumor-to-background contrast (tumor/muscle = 53.5 and 76.2 at 1 h and 2 h, resp.). No significant defluorination from [^18^F]**8** uptake at bone was observed up to 2 h after injection. Furthermore, inhibition study showed that the excess amount (10 mg/kg) of c(RGDyK) peptide significantly blocked [^18^F]**8** uptake in the tumor (tumor/muscle = 8.2 and 6.3 at 1 h and 2 h, resp.) (Figure 4). These *in vivo* imaging studies indicated that ^18^F-labelled RGD peptide via photoinduced 1,3-dipolar cycloaddition could successfully visualize tumor *in vivo* through integrin *α*_v_*β*_3_.

## 3. Conclusion

Our results suggest that ^18^F-labelled RGD([^18^F]**8**) radiotracer is useful for monitoring tumor response in angiogenesis research. The development of novel radiolabelling method for diagnosis of various diseases including cancer has benefit for mild conditions as an important approach to obtain accurate imaging. Photoinduced 1,3-dipolar cycloaddition using ^18^F-radioisotope is an efficient radiolabelling strategy to prepare molecular imaging probes. It could be applied as a bioorthogonal approach for chemical modification in biomedical research. Further study is required on the application of such photoinducible radiolabelling strategy based on its high availability and excellent chemoselectivity.

## 4. Materials and Methods

Benzenesulfonyl hydrazide, sodium nitrite, aniline, 4-fluorobenzoic acid, *N*,*N*′-dicyclohexylcarbodiimide (DCC), 4-dimethylaminopyridine (DMAP), 2-allyloxyethanol, dimethylaminobenzoyl chloride, methyl trifluoromethanesulfonate (MeOTf), triethylamine (TEA), *N*-hydroxysuccinimide, 1-ethyl-3-3-dimethylaminopropylcarbodiimide (EDC), 4-formylbenzoic acid, and DIPEA (*N*,*N*′-diisopropylethylamine) of ACS grade were purchased from Sigma-Aldrich (St. Louis, MO, USA). c(RGDyK) was purchased from Futurechem (Seoul, Korea). All chemicals were used without further purification unless otherwise noted. [^18^F]Fluoride ion was produced from cyclotron (KIRAMS, South Korea) with 13 MeV proton irradiation. Flash column chromatography was performed using 230–400 mesh silica gel (Merck KGaA, Darmstadt, Germany). Radio-TLC was analyzed using a System 200 Imaging Scanner (BioScan, CA, USA). Purification was performed with a spectra system SCM100 degasser, a P4000 pump, and a UV-Vis 3000 detector (Thermo Scientific, Waltham, MA, USA). Absorbance was monitored at 214 nm with a C18 reverse-phase HPLC column (250 mm × 10 mm, 5 *µ*m, Thermo Scientific, MA, USA). ChromQuest 4.2 software was used to record chromatograms. NMR spectra were recorded with a 600 MHz FT-NMR (JNM-EAC600, JEOL, Tokyo, Japan). High-resolution mass spectra (*m*/*z*) were recorded with electron ionization (EI, DFS (Thermo Scientific, Germany)) and fast atom bombardment (FAB, JMS-700 (JEOL, Tokyo, Japan)) at KBSI (Seoul, Korea).

### 4.1. Synthesis of 2-(Allyloxy)ethyl 4-(dimethylmino)benzoate

2-Allyloxyethanol (2.0 mL, 18.7 mmol) was added to 4-*N*,*N*-dimethylaminobenzoyl chloride (0.5 g, 2.72 mmol) and triethylamine (0.76 mL, 5.44 mmol) in 10 mL of dichloromethane in a flame-dried flask with nitrogen stream. The reaction mixture was stirred for two hours at room temperature. The reaction mixture was then diluted with 50 mL of ethyl acetate and 30 ml of water. The product of organic layer was washed twice with 30 mL of water, and the organic layer was washed with 50 mL of brine. The organic layer was dried over sodium sulfate, evaporated, and purified with flash column chromatography (ethyl acetate : hexane = 1 : 5) to obtain a yellow oil product with yield of 55% (373.7 mg). ^1^H NMR (600 MHz, CDCl_3_) data were *δ* (ppm) = 7.91 (m, 2H), 6.63 (m, 2H), 5.90 (m, 1H), 5.29 (m, 1H), 5.18 (m, 1H), 4.40 (m, 2H), 4.05 (m, 2H), 3.74 (m, 2H), and 3.01 (s, 6H). ^13^C NMR (150 MHz, CDCl_3_) data were *δ* (ppm) = 167.00, 153.39, 134.68, 131.48, 117.29, 116.95, 110.75, 72.22, 68.37, 63.57, and 40.14. FAB MS calculated for C_14_H_19_O_3_N_1_Na_1_ was 272.1257*m*/*z* [M + Na]. It was also found to be 272.1257.

### 4.2. Synthesis of 4-((2-(Allyloxy)ethoxy)carbonyl)-*N*,*N*,*N*-trimethylbenzenaminium (**1**)

To a solution of 2-(allyloxy)ethyl 4-(dimethylamino)benzoate (0.3 g, 1.20 mmol) dissolved in 2 mL of dichloromethane, methyl trifluoromethanesulfonate (0.27 mL, 2.40 mmol) was added. The mixture was stirred for two hours with nitrogen stream at room temperature. The reaction mixture was evaporated, and the resulting crude mixture was dissolved in 0.5 mL of ethanol. The product was crystallized with 100 mL of diethyl ether and dried to obtain a compound with a yield of 38% (122 mg). ^1^H NMR (600 MHz, CDCl_3_) data were *δ* (ppm) = 8.26 (d, *J* = 8.4 Hz, 2H), 7.92 (d, *J* = 9 Hz, 2H), 5.94–5.88 (m, 1H), 5.29 (d, *J* = 17.1 Hz, 1H), 5.20 (d, *J* = 10.5, 1H), 4.51 (t, *J* = 9.6 Hz, 2H), 4.06 (t, *J* = 2.4 Hz, 1H), 4.05 (t, *J* = 2.4 Hz, 1H), and 3.78 (s, 9H). ^13^C NMR (150 MHz, CDCl_3_) data were *δ* (ppm) = 164.65, 150.42, 134.50, 132.13, 131.34, 120.50, 116.09, 71.64, 67.67, 64.58, and 56.39. FAB MS calculated for C_15_H_22_O_3_N_1_ was 264.1594 *m*/*z* [M]. It was found to be 264.1594.

### 4.3. Synthesis of 2-(Allyloxy)ethyl 4-[^18^F]fluorobenzoate ([^18^F]**2**)

Aqueous [^18^F]fluoride of 0.76 GBq was added to an open glass reaction vessel containing 1 mL of Kryptofix 2.2.2 of solution (5 mg of Kryptofix 2.2.2 in 800 *µ*L of acetonitrile and 1.5 mg of K_2_CO_3_ in 200 *µ*L of H_2_O). Azeotropic distillation was carried out to remove water at 95°C with a nitrogen stream. This procedure was repeated 4-5 times by further addition of 20 *µ*L anhydrous acetonitrile until a white powder was obtained. The resulting K[^18^F]F complex was dissolved in acetonitrile (200 *µ*L). The resulting K[^18^F]F solution was transferred to a reaction vial containing 4-((2-(allyloxy)ethoxy)carbonyl)-*N*,*N*,*N*-trimethylbenzenaminium (2 mg) and heated at 100°C with stirring for 20 min. At the end of the reaction, ^18^F-labelled desired compound showed a yield of 79% on radio-TLC using 1 : 5 mixture of ethyl acetate-hexane as developing solvent. We used crude mixture of [^18^F]**2** in the next step for photoinducible click reaction without purification.

### 4.4. Synthesis of 2-(Allyloxy)ethyl 4-Fluorobenzoate (**2**)

Synthesis of 2-(allyloxy)ethyl 4-fluorobenzoate was carried out according to the published method of Vaidyanathan et al. [33] with slight modifications. Briefly, 2-allyloxyethanol (0.76 ml, 0.71 mmol) was added to a mixture of 4-fluorobenzoic acid (0.1 g, 0.71 mmol), DCC (0.15 g, 0.71 mmol), and DMAP (0.6 mg, 8.0 mmol) in 4 mL of ethyl acetate with a N_2_ stream in a flask dried with a heat gun. The reaction mixture was stirred at room temperature overnight, and a white precipitate was filtrated out. After removing the precipitate, the residual reaction mixture was evaporated and purified by flash column chromatography (ethyl acetate : hexane = 1 : 5), resulting in a colorless oil with a yield of 42% (67.6 mg). 1H NMR (600 MHz, CDCl_3_) data were *δ* (ppm) = 8.10 (m, 2H), 7.13 (t, *J* = 8.6 Hz, 2H), 5.96 (m, 1H), 5.33 (dd, *J* = 38.6 Hz, 16.8 Hz, 10 Hz, 2H), 4.49 (t, *J* = 5 Hz, 2H), 4.08 (d, *J* = 6 Hz, 2H), and 3.79 (t, *J* = 4.8 Hz, 2H). ^13^C NMR (100 MHz, CDCl_3_): *δ* (ppm) = 167.2, 165.7, 164.7, 134.5, 132.4, 132.3, 126.5, 117.6, 115.7, 115.5, 68.1, and 64.4. FAB MS calculated for C_12_H_14_O_3_F_1_ was 225.0921 *m*/*z* [M + H]. It was found to be 225.0921.

### 4.5. Synthesis of 5-(4-Methoxyphenyl)-2-phenyl-2*H*-tetrazole (**3**)

For the preparation of phenylsulfonylhydrazone [29], *p*-anisaldehyde (0.68 g, 5 mmol) was dissolved in 50 mL of ethanol and mixed with benzensulfonylhydrazide (0.86 g, 25 mmol). The mixture was stirred at room temperature for 30 min. After addition of 100 mL water, a white precipitate formed. It was separated with a filter. Diazonium salt solution was prepared by adding NaNO_2_ (0.35 g, 5 mmol) into 2 mL of water. It was dropped into cooled aniline (0.47 g, 5.0 mmol) dissolved in 8 mL of water/ethanol (1 : 1) and 1.3 mL of concentrated hydrochloric acid (∼36%). Phenylsulfonylhydrazone was dissolved in 30 mL of pyridine. Diazonium salt was then added dropwise with stirring at −10°C. A red precipitate formed after addition of 250 mL of 3NHCl. The precipitate was then collected and extracted with chloroform and water. The organic layer was dried and subjected to flash column chromatography on a silica gel (dichloromethane : ethyl acetate = 50 : 1). A red solid was obtained with a yield of 30% (382 mg).1H NMR (600 MHz, CDCl_3_) data were *δ* (ppm) = 8.21 (d, *J* = 8.8 Hz, 4H), 7.60 (t, *J* = 8 Hz, 2H), 7.51 (t, *J* = 7.4 Hz, 1H), 7.10 (d, *J* = 8.8 Hz, 2H), and 3.90 (s, 3H). ^13^C NMR (100 MHz, chloroform-d_3_): *δ* (ppm) = 129.8, 129.6, 128.7, 119.9, 114.5, 55.6, 29.9, 22.9, and 14.3. FAB MS calculated for C_14_H_13_O_1_N_4_ was 253.1084 *m*/*z* [M + H]. It was found to be 253.1084.

### 4.6. Synthesis of 2-((3-(4-Methoxyphenyl)-1-phenyl-4,5-dihydro-1H-pyrazol-4-yl)methoxy)ethyl 4-Fluorobenzoate (**4**)

Photoinducible 1,3-dipolar cycloaddition was performed between **2** (0.1 g, 0.4 mmol) and **3** (0.18 g, 0.79 mmol) in 16 mL of mixture of acetonitrile/PBS (50/50). The reaction mixture was irradiated with 302 nm UV lamp for 5 min, 10 min, 20 min, 30 min, 1 h, and 2 h with stirring at room temperature. After that, the mixture was extracted with chloroform and water. The organic layer was then dried. The crude mixture was then purified by flash column chromatography on silica gel (dichloromethane : ethyl acetate = 50 : 1) to obtain a product with a yield of 63% (112.4 mg). 1H NMR (600 MHz, acetonitrile-d_3_): *δ* (ppm) = 8.02 (dd, *J* = 8.7 Hz, 5.4 Hz, 2H), 7.79 (dd, *J* = 8.1 Hz, 2.4 Hz, 2H), 7.67 (dd, *J* = 8.4 Hz, 1.2 Hz, 2H), 7.45 (t, *J* = 7.5 Hz, 2H), 7.40 (tt, *J* = 7.5 Hz, 1.2 Hz, 1H), 7.20 (t, *J* = 8.7 Hz, 2H), 6.98 (dd, *J* = 6.6 Hz, 22.4 Hz, 2H), 6.81 (s, 1H), 4.58 (s, 2H), 4.44 (t, *J* = 4.8 Hz, 2H), 3.84 (m, 4H), and 3.82 (s, 3H). ^13^C NMR (151 MHz, acetonitrile-d_3_): *δ* (ppm) = 166.6, 165.2, 159.8, 151.0, 140.7, 139.9, 132.2, 129.2, 127.7, 126.8, 125.8, 124.1, 115.7, 115.6, 114.4, 105.8, 67.9, 64.2, 62.9, 55.0, and 16.6. FAB MS calculated for C_26_H_25_O_4_N_0_F_1_ was 448.1793 *m*/*z* [M]. It was found to be 448.1790.

### 4.7. Synthesis of 2-((3-(4-Methoxyphenyl)-1-phenyl-4,5-dihydro-1H-pyrazol-4-yl)methoxy)ethyl 4-[^18^F]fluorobenzoate ([^18^F]**4**)

Photoinducible 1,3-dipolar cycloaddition was performed with **3** (1 mg, 3.96 mmol) and 2-(allyloxy) ethyl 4-[^18^F]fluorobenzoate ([^18^F]**2**) in 0.2 mL of acetonitrile-phosphate buffer = 1 : 1. The reaction mixture was irradiated with 302 nm UV lamp for 30 min with stirring. For purification, the reaction mixture was immediately loaded into RP-HPLC (A = 0.1% TFA in water/B = 0.1% TFA in acetonitrile, 254 nm, 3.0 mL/min) followed by gradient purification (isocratic flow with 10% B for 5 min, gradient increase from 10% → 100% B for 25 min, and maintaining the flow with 100% B for 10 min). Retention time of the desired compound was 27 min. RP-HPLC was performed for the collected peak for identification using nonlabelled standard compound. Decay-corrected radiochemical yield of [^18^F]**4** was 36% including HPLC purification and synthesis time was 58 min.

### 4.8. Synthesis of 4-(2-Phenyl-2H-tetrazol-5-yl)benzoate (**5**)

Compound **5** was prepared by a previously reported procedure [29]. To a flask containing compound 4-formylbenzoic acid (2.25 g, 15.0 mmol), ethanol (50 mL) was added. Benzoylsulfonohydrazide (2.58 g, 75.0 mmol) was added to the above solution. A white precipitate formed after addition of 150 mL water. It was collected in a funnel. The white solid was dissolved in 90 mL pyridine to give solution A. A solution of NaNO_2_ (1.04 g, 15.0 mmol) in 6 mL water was added dropwise to a cooled mixture of aniline (1.40 g, 15.0 mmol) dissolved in 24 mL water-ethanol (1 : 1) and 4 mL concentrated HCl to give solution B. Solution B was added slowly to solution A cooled with an ice bath. The reaction mixture was then extracted with ethyl acetate (3 × 30 mL). A precipitate formed after adding 750 mL 3NHCl to the combined organic layers. It was collected and dried. The desired product was analyzed by LC/MS/MS and ^1^H/^13^C NMR. 1H NMR (600 MHz, chloroform-d_3_): *δ* (ppm) = 8.27 (d, *J* = 4.2 Hz, 2H), 8.14 (t, *J* = 8.4 Hz, 4H), 7.68 (t, *J* = 8.4 Hz, 2H), and 7.62 (t, *J* = 7.2 Hz, 1H). ^13^C NMR (100 MHz, chloroform-d_3_): *δ* (ppm) = 167.2, 164.3, 136.6, 133.3, 130.9, 130.8, 127.3, and 120.5. LC-mass/HRMS (*m*/*z*): [M + H]^+^ calculated for C_14_H_11_N_4_O_2_ was 267.0884; it was found to be 267.0885.

### 4.9. Synthesis of 2,5-Dioxopyrrolidin-1-yl 4-(2-phenyl-2H-tetrazol-5-yl)benzoate (**6**)

N-hydroxysuccinimide (NHS, 86.5 mg, 0.75 mmol) was added to a mixture of 1-ethyl-3-3-dimethylaminopropylcarbodiimide (EDC, 116.7 mg, 0.75 mmol) and 4-(2-phenyl-2H-tetrazole-5-yl)-benzoic acid (100 mg, 0.38 mmol) in 5 mL acetonitrile followed by incubation at room temperature overnight with a stream of N_2_ gas. The reaction mixture was then diluted with 100 mL of CH_2_Cl_2_ and water. The organic layer was washed three times with 100 mL of water followed by wash with 100 mL of brine once. The organic layer was dried over sodium sulfate and then evaporated. The resulting crude mixture was purified with flash column chromatography (ethyl acetate : hexane = 1 : 1). An orange powder was obtained with a yield of 21.9% (30 mg). The product was analyzed by LC/MS/MS and ^1^H/^13^C NMR. ^1^H NMR (600 MHz, CDCl_3_): *δ* (ppm) = 8.42 (d, *J* = 9 Hz, 2H), 8.29 (d, *J* = 9 Hz, 2H), 8.21 (d, *J* = 7.8 Hz, 2H), 7.59 (t, *J* = 15 Hz, 2H), 7.53 (t, *J* = 15.6 Hz, 1H), and 2.94 (s, 9H). ^13^C NMR (151 MHz, dichloromethane-d_2_): *δ* (ppm) = 169.8, 164.5, 162.1, 131.7, 130.6, 130.4, 127.9, 120.5, 34.5, and 26.3. LC-mass/HRMS (*m*/*z*): [M + H]^+^ calculated for C_18_H_14_N_5_O_4_ was 364.1047; it was found to be 364.1040.

### 4.10. Synthesis of 2,5-Dioxopyrrolidin-1-yl 4-(2-phenyl-2H-tetrozol-5-yl)benzoate-RGD Conjugate (**7**)

Cyclic Arg-Gly-Asp-D-Tyr-Lys (cRGDyK, 5.8 mg, 0.006 mmol) was added to a mixture of **6** (5 mg, 0.01 mmol) and *N*,*N*′diisopropylethylamine -(DIPEA, 1.8 mg, 0.01 mmol) in 1 mL of dimethylformamide (DMF) followed by incubation at room temperature for two hours with a stream of N_2_ gas. The mixture was purified using RP-HPLC. Flow rate was set at 2.5 ml/min. The mobile phase consisted of solvent A (0.1% trifluoroacetic acid in water) and solvent B (0.1% trifluoroacetic acid in acetonitrile). Gradient details were as follows: 0–5 min, 1% B; 5–30 min, 20% B; 30–45 min, 70% B; and 45–60 min, 100% B. Retention time of the product was 43 min. The product was analyzed by LC/MS/MS. LC-mass/HRMS (*m*/*z*): [M + H]^+^ calculated for C_41_H_50_N_13_O_9_ was 868.3855; it was found to be 868.3848.

### 4.11. Synthesis of FB-PEGhc-RGD (**8**)

A photoinducible 1,3-dipolar cycloaddition was performed between 2,5-dioxopyrrolidin-1-yl 4-(2-phenyl-2H-tetrozol-5-yl)benzoate-RGD (2, 1.0 mg, 0.001 mmol) and 2-(allyloxy)ethyl 4-fluorobenzoate (3.8 mg, 0.017 mmol) in 200 *µ*L of mixture of acetonitrile/PBS (50/50). The reaction mixture was irradiated with 302 nm UV lamp for 30 min with stirring at room temperature. The mixture was purified using RP-HPLC. The flow rate was set at 3 mL/min with mobile phase consisting of solvent A (0.1% trifluoroacetic acid in water) and solvent B (0.1% trifluoroacetic acid in acetonitrile). Gradient details were as follows: 0 min, 30% B; 0–22 min, 100% B; and 22–23 min, 30% B. The retention time was 8.5 min. The desired product was analyzed by LC/MS/MS. LC-mass/HRMS (*m*/*z*): [M + H]^+^ calculated for C_53_H_63_FN_11_O_12_ was 1064.4642; it was found to be 1064.4287.

### 4.12. Synthesis of ^18^FB-PEGhc-RGD ([^18^F]**8**)

Photoinducible 1,3-dipolar cycloaddition was performed by mixing 2,5-dioxopyrrolidin-1-yl 4-(2-phenyl-2H-tetrozol-5-yl)benzoate-RGD (1 mg) and 2-(allyloxy)ethyl 4-[^18^F]fluorobenzoate in 0.2 ml of acetonitrile-phosphate buffer at 1 : 1. The reaction mixture was irradiated with 302 nm UV lamp for 30 min with stirring. The reaction mixture was purified using RP-HPLC. The flow was at 3 mL/min. Mobile phase consisted of solvent A (0.1% trifluoroacetic acid in water) and solvent B (0.1% trifluoroacetic acid in acetonitrile). Gradient details were as follows: 0 min, 30% B; 0–22 min, 100% B; and 22–23 min, 30% B. Retention time of the desired compound was 8.5 min. Decay-corrected radiochemical yield of [^18^F]**8** was 10–12% (*n*=4), and total synthesis time was 134 min including HPLC purification and reformulation.

### 4.13. Tumor Cell Uptake Assay

U87MG human glioma cells were maintained and subcultured every other day in Roswell Park Memorial Institute (RPMI) 1640 media supplemented with 10% fetal bovine serum and 1% penicillin-streptomycin at 37°C in 5% CO_2_ and 95% air environment. The cells were seeded into 24-well plates at density of 5 × 10^4^ cells/well and cultured overnight. The cells were rinsed once with PBS followed by addition of [^18^F]**8** (∼0.33 MBq/well) or RGD (100 *μ*M/well) to cultured wells in quadruplicate. After incubation at 37°C for 5, 15, 30, 60, and 120 min, cells were rinsed twice with cold PBS and harvested after treatment with TrypLE. The cells were collected in measurement tubes for counting. Finally, radioactivity of the cells was measured using a gamma counter. All experiments were performed in quadruplicate. Results are expressed as mean ± SD.

### 4.14. *In Vivo* Experiments

#### 4.14.1. Tumor Models

All experimental protocols with animals were performed in compliance with the policies and procedures of the Institutional Animal Care and Use Committee of Chonbuk National University (Jeonju, Korea). Four male SD rats were purchased from Orient-Bio (Seoul, Korea) at 13 weeks of age. They were injected subcutaneously (s.c.) in the right flank with 1 × 10^7^ RR1022 fibrosarcoma cells suspended in 150 *µ*L RPMI 1640 medium. When tumors reached 0.8–1.0 cm in diameter (7 d after inoculation), rats were used for microPET imaging experiment.

#### 4.14.2. MicroPET Studies

MicroPET scans and image data analysis were performed using a Biograph TruePoint 40 PET/CT scanner (Siemens Medical Solutions, Knoxville, TN, USA). Rat bearing RR1022 tumor was tail-vein injected with 5.1 MBq of [^18^F]**8** under zoletil anesthesia (mg/kg). Ten-minute static PET images were then acquired at two time points (1 h and 2 h) after injection. CT scan was obtained first by a continuous spiral technique (120 kVp, 200 Ma, 0.5 s rotation time). A PET scan was then acquired in 3-dimensional mode at 15 minutes per bed position. Obtained PET data were reconstructed iteratively using an ordered-subset expectation maximization algorithm. Initial CT data were used for attenuation correction. For receptor-blocking experiment, c(RGDyK) (10 mg/kg) was coinjected with 5.4 MBq of [^18^F]**8** to RR1022 tumor rat. At 1 h and 2 h after injection, ten-minute static microPET scans were acquired. Assessment of tracer distribution in tumor tissue was expressed as tumor-to-muscle (T/M) ratio, dividing the mean activity within the ROI of the tumor by the mean activity within thigh muscle ROI.

## References

[1] Herschman H. R. (2003). Molecular imaging: looking at problems, seeing solutions. *Science*.

[2] Pimlott S. L., Sutherland A. (2011). Molecular tracers for the PET and SPECT imaging of disease. *Chemical Society Reviews*.

[3] Massoud T. F., Gambhir S. S. (2003). Molecular imaging in living subjects: seeing fundamental biological processes in a new light. *Genes & Development*.

[4] Phelps M. E. (2000). Positron emission tomography provides molecular imaging of biological processes. *Proceedings of the National Academy of Sciences*.

[5] Saraste A., Nekolla S. G., Schwaiger M. (2009). Cardiovascular molecular imaging: an overview. *Cardiovascular Research*.

[6] McGeer P., McGeer E. (1995). The inflammatory response system of brain: implications for therapy of Alzheimer and other neurodegenerative diseases. *Brain Research Reviews*.

[7] Reivich M., Kuhl D., Wolf A. (1977). Measurement of local cerebral glucose metabolism in man with 18F-2-fluoro-2-deoxy-d-glucose. *Acta Neurologica Scandinavica: Supplementum*.

[8] Gambhir S. S. (2002). Molecular imaging of cancer with positron emission tomography. *Nature Reviews Cancer*.

[9] Ametamey S. M., Honer M., Schubiger P. A. (2008). Molecular imaging with PET. *Chemical Reviews*.

[10] O’Hagan D. (2008). Understanding organofluorine chemistry: an introduction to the C-F bond. *Chemical Society Reviews*.

[11] Vaidyanathan G., Zalutsky M. R. (1992). Labeling proteins with fluorine-18 using N-succinimidyl 4-[^18^F]fluorobenzoate. *International Journal of Radiation Applications and Instrumentation: Part B: Nuclear Medicine and Biology*.

[12] Guhlke S., Coenen H. H., Stocklin G. (1994). Fluoroacylation agents based on small nca [F-18]fluorocarboxylic acids. *Applied Radiation and Isotopes*.

[13] Johnström P., Clark J. C., Pickard J. D., Davenport A. P. (2008). Automated synthesis of the generic peptide labelling agent *N*-succinimidyl 4-[^18^F]fluorobenzoate and application to ^18^F-label the vasoactive transmitter urotensin-II as a ligand for positron emission tomography. *Nuclear Medicine and Biology*.

[14] Toretsky J., Levenson A., Weinberg I. N., Tait J. F., Uren A., Mease R. C. (2004). Preparation of F-18 labeled annexin V: a potential PET radiopharmaceutical for imaging cell death. *Nuclear Medicine and Biology*.

[15] Vaidyanathan G., Zalutsky M. R. (2006). Synthesis of *N*-succinimidyl 4-[^18^F]fluorobenzoate, an agent for labeling proteins and peptides with ^18^F. *Nature Protocols*.

[16] Kuyhnast B., Bruin B., Hinnen F., Tavitian B., Dolle F. (2004). Design and synthesis of a new [^18^F]fluoropyridine-based haloacetamide reagent for the labeling of oligonucleotides: 2-bromo-*N*-[3-(2-[18F]fluoropyridin-3-yloxy)propyl]acetamide. *Bioconjugate Chemistry*.

[17] von Guggenberg E., Sader J. A., Wilson J. S. (2009). Automated synthesis of an ^18^F-labelled pyridine-based alkylating agent for high yield oligonucleotide conjugation. *Applied Radiation and Isotopes*.

[18] Kühnast B., Dollé F., Vaufrey F., Hinnen F., Crouzel C., Tavitian B. (2000). Fluorine-18 labeling of oligonucleotides bearing chemically-modified ribose-phosphate backbones. *Journal of Labelled Compounds and Radiopharmaceuticals*.

[19] Kuhnast B., Hinnen F., Hamzavi R. (2005). Fluorine-18 labelling of PNAs functionalized at their pseudo-peptidic backbone for imaging studies with PET. *Journal of Labelled Compounds and Radiopharmaceuticals*.

[20] Huisgen R., Padwa A. (1984). *1,3-Dipolar Cycloaddition Chemistry*.

[21] Kolb H. C., Finn M. G., Sharpless K. B. (2001). Click chemistry: diverse chemical function from a few good reactions. *Angewandte Chemie International Edition*.

[22] Bock V. D., Hiemstra H., van Maarseveen J. H. (2006). Cu(I) catalyzed alkyne–azide “click” cycloadditions from a mechanistic and synthetic perspective. *European Journal of Organic Chemistry Banner*.

[23] Marik J., Sutcliffe J. L. (2006). Click for PET: rapid preparation of [^18^F]fluoropeptides using Cu(I) catalyzed 1,3-dipolar cycloaddition. *Tetrahedron Letters*.

[24] Kim D. H., Choe Y. S., Kim B. T. (2010). A ^18^F-labeled glucose analog: synthesis using a click labeling method and in vivo evaluation. *Applied Radiation and Isotopes*.

[25] Lee C. M., Jeong H. J., Kim D. W., Sohn M. H., Lim S. T. (2012). The effect of fluorination of zinc oxide nanoparticles on evaluation of their biodistribution after oral administration. *Nanotechnology*.

[26] Bouvet V., Wuest M., Wuest F. (2011). Copper-free chemistry with the short-lived positron emitter fluorine-18. *Organic & Biomolecular Chemistry*.

[27] Campbell-Verduyn L. S., Mirfeizi L., Schoonen A. K., Dierckx R. A., Elsinga P. H., Feringa B. L. (2011). Strain-promoted copper-free “click” chemistry for ^18^F radiolabeling of bombesin. *Angewandte Chemie International Edition*.

[28] Wang Y., Vera C. I., Lin Q. (2007). Convenient synthesis of highly functionalized pyrazolines via mild, photoactivated 1,3-dipolar cycloaddition. *Organic Letters*.

[29] Song W., Wang Y., Qu J., Madden M. M., Lin Q. (2008). A photoinducible 1,3-dipolar cycloaddition reaction for rapid, selective modification of tetrazole-containing proteins. *Angewandte Chemie International Edition*.

[30] Lim R. K. V., Lin Q. (2011). Photoinducible bioorthogonal chemistry: a spatiotemporally controllable tool to visualize and perturb proteins in live cells. *Accounts of Chemical Research*.

[31] Sheldrake H. M., Patterson L. H. (2009). Function and antagonism of β integrins in the development of cancer therapy. *Current Cancer Drug Targets*.

[32] Temming K., Schiffelers R. M., Molema G., Kok R. J. (2005). RGD-based strategies for selective delivery of therapeutics and imaging agents to the tumour vasculature. *Drug Resistance Updates*.

[33] Vaidyanathan G., White B. J., Zalutsky M. R. (2009). Propargyl 4-[^18^F]fluorobenzoate: a putatively more stable prosthetic group for the fluorine-18 labeling of biomolecules via click chemistry. *Current Radiopharmaceuticalse*.

